# Symposium on Anaemia

**Published:** 1917-06

**Authors:** 


### SYMPOSIUM ON ANAEMIA.

Dr. L. NAPOLEON BOSTON, Philadelphia.

1. To the ultra scientific physician the pathologic classification of anaemia appeals most strongly. To the practitioner the clinical classification is the far more significant.

2. I have employed numerous proportions of iron for many years, and am frank to admit that in the majority of cases, the results obtained are less favorable than those given by the use of other remedies. Food rich in iron, that is available through digestion, are preferable.

3. Whenever the cause of the anaemia present can be ascertained the treatment is governed by such data. In those cases not the result of advancing cardiac, renal, hepatic disease, or carcinoma, excellent results follow the use of readily digested foods, meat juices; and special attention to the gastric intestinal tract.

4. A broth that I use in the treatment of anaemia is as follows:

(a) 1—One pound of ground lean beef.
2—Two quarts of water.
2—One raw potato ground or crushed.
4—One or two stalks of crushed celery.

Mix this together, and allow it to stand in a cool place for from eight to twelve hours.

(b) Place over the fire, and allow it to steep slowly until the volume of liquid approximates one quart.

(c) Strain through a towel, and place on ice.

(d) Give the patient six to eight ounces of this broth three times per day, heating for each feeding, and seasoning to suit the patient's taste.

This together with the administration of red bone marrow proved to me in the treatment of sixty cases of anaemia that the red blood corpuscles, and the hemoglobin were more rapidly increased by this method, together with the fact that the patients were always fed liberally of green fruit and vegetables, than by any other method I have yet employed.

---

Dr. JUDSON DALAND, Philadelphia.

I prefer not to express an opinion as regards the best classification of the Anaemias, but would like to dwell strongly upon the importance of the opinion that pernicious anaemia is usually a secondary anaemia, and that often the primary cause cannot be discovered; but failure to discover the cause does not entitle the anaemia to be considered as primary.

Upon the whole, I have been absolutely unable to convince myself that in any ordinary anaemia any preparation of iron is of any special importance; and not infrequently I order an excess of iron-containing food, more especially spinach. In my judgment, anaemia, independent of iron starvation cannot be benefitted by the administration of iron. So far as I have been able to ascertain, most of the iron administered by the mouth escapes by way of the bowels.

Successful treatment of anaemia depends upon first, the discovery of the cause, and second, ability to remove it. A common and frequent unsuspected cause of chloro-anaemia is due to absorption of decomposition products in the colon, and some times to results of fermentation of colonic contents. Another common cause is insufficient intake of oxygen, due to insufficient respiration; and still another is an unrecognized, latent, chronic septic focus, which may be anywhere, but which is more usually in the mouth, tonsils or nasal cavities. When the organism is the streptococcus haemolyticus, very severe anaemia may occasionally be the consequence.

---

Dr. ANTHONY BASSLER, New York City.

Answering the five questions contained in your letter of request, I offer the following:

1. "What do you consider the best classification of anaemias, based on clinical and pathological data?"

There has been little advance in the classification of anaemias. The best at present is still the old clinical one. This is the simplest and the most practical, although it is far from pathological in an etiological sense or even in tissue change.

2. "In what kind of anaemia and with what proportion of success is the administration of iron indicated?"

In the secondary anaemias of all types. In the primaries of all grades (and there are many up to the border-line cases of true dyscrasis). But when true pernicious anaemia or the true leukemias exist, its use is not of considerable value.

3. "Do you prefer inorganic, artificial organic forms of iron or those naturally present in foods?"

In the mild and moderate degrees of secondary anaemia organic iron and heavy iron-bearing foods (meats, yolk of eggs, green chicory, cabbage, spinach) answer. In the more severe, the inorganic plus the ferrous types of foods. No form of foods answer in the leukemias, a generous diet answers as well as any and is the best one can advise.

4. "Mention any special preparations found useful. What kinds of anaemia are independent of iron starvation, that is, develop on a diet sufficiently rich in iron and cannot be relieved by giving iron in any form?"

In the secondary anaemias of severe grades fresh Blaud mass, tincture ferri chloride and pyrophosphate of iron. For the moderate and slight grades, liquid ferri albuminate, liquid ferri and manganese peptonate, ovoferrin, reduced iron. For hypodermic administration, green iron citrate and iron cacodylate in all grades. In the leukemias the arsenic preparations answer best, the wisest plan being to use the hypodermic method and solutions of iron arsenite, Fowler's solution, and odium Cacodylate. The anaemias independent of iron starvation are pernicious anaemia and both of the leukemias.

5. Please state your views as to absorption, assimilation and elimination of iron, with special reference to the nature of anaemia as a failure of assimilation or an actual destruction of hemoglobin."

There are instances of anaemia in which the iron of foods are rendered impossible of resorption by it being acted upon by free sulphur gas ($H_2$ $S_2$) in the bowel and its change into insoluble sulphide. Here the hypodermic administration works best. I do not see how there could be a disorder of over elimination of iron from the general circulation—excepting, of course, a frank haemorrhage for any reason. Iron from the circulation is only eliminated by the pigments of bile, urine, etc., and this demand of iron is small, even in greatest activity of function. The main point in therapeutics of anaemia is to get the iron into the body in combined state with the tissue. At best the body takes up iron slowly, and when in a real blood dyscrasia exists, like pernicious anaemia the slow assimilation cannot meet the demands for hemoglobin content. Here, however, the main trouble is in the body. According to its degree, the hemopoetic apparatus does not replenish the constant utilization and normal breaking down of erythrocytes. The practical point is not iron deficiency, but deficiency of erythrocytic body formation in which iron is only one part of the complicated organic tissue chemistry. It is being taught and believed that pernicious anaemia is a degenerative disease of the erythrocytes—it is more logical to believe that the blood condition is one of failure of production of new cells to meet those constantly breaking down in normal physiology.

